# The Expression Patterns and Roles of Lysyl Oxidases in Aortic Dissection

**DOI:** 10.3389/fcvm.2021.692856

**Published:** 2021-07-07

**Authors:** Xin Yi, Yi Zhou, Yue Chen, Xin Feng, Chang Liu, Ding-Sheng Jiang, Jing Geng, Xiaoyan Li, Xuejun Jiang, Ze-Min Fang

**Affiliations:** ^1^Department of Cardiology, Renmin Hospital of Wuhan University, Wuhan, China; ^2^Cardiovascular Research Institute, Wuhan University, Wuhan, China; ^3^Hubei Key Laboratory of Cardiology, Wuhan, China; ^4^Division of Cardiothoracic and Vascular Surgery, Tongji Hospital, Tongji Medical College, Huazhong University of Science and Technology, Wuhan, China

**Keywords:** lysyl oxidase, aortic dissection, LOX, MMP2, metformin, losartan

## Abstract

**Background:** Lysyl oxidases (LOXs), including LOX, LOXL1, LOXL2, LOXL3, and LOXL4, catalyze the formation of a cross-link between elastin (ELN) and collagen. Multiple LOX mutations have been shown to be associated with the occurrence of aortic dissection (AD) in humans, and LOX-knockout mice died during the perinatal period due to aortic aneurysm and rupture. However, the expression levels and roles of other LOX members in AD remain unknown.

**Methods:** A total of 33 aorta samples of AD and 15 normal aorta were collected for LOXs mRNA and protein levels detection. We also analyzed the datasets of AD in GEO database through bioinformatics methods. LOXL2 and LOXL3 were knocked down in primary cultured human aortic smooth muscle cells (HASMCs) via lentivirus.

**Results:** Here, we show that the protein levels of LOXL2 and LOXL3 are upregulated, while LOXL4 is downregulated in AD subjects compared with non-AD subjects, but comparable protein levels of LOX and LOXL1 are detected. Knockdown of LOXL2 suppressed MMP2 expression, the phosphorylation of AKT (p-AKT) and S6 (p-S6), but increased the mono-, di-, tri-methylation of H3K4 (H3K4me1/2/3), H3K9me3, and p-P38 levels in HASMCs. These results indicate that LOXL2 is involved in regulation of the extracellular matrix (ECM) in HASMCs. In contrast, LOXL3 knockdown inhibited PCNA and cyclin D1, suppressing HASMC proliferation. Our results suggest that in addition to LOX, LOXL2 and LOXL3 are involved in the pathological process of AD by regulating ECM and the proliferation of HASMCs, respectively. Furthermore, we found that LOXL2 and LOXL4 was inhibited by metformin and losartan in HASMCs, which indicated that LOXL2 and LOXL4 are the potential targets that involved in the therapeutic effects of metformin and losartan on aortic or aneurysm expansion.

**Conclusions:** Thus, differential regulation of LOXs might be a novel strategy to prevent or treat AD.

## Background

Aortic dissection (AD) is a life-threatening condition, and the annual incidence of AD is estimated to be 3.8–8.8 per 100,000 persons (1–3). Patients with acute Stanford type A AD (TAAD) who do not receive proper operation have a mortality rate of up to 50% within the first 48 h, and surgery is the main treatment of choice due to the lack of effective conservative treatment strategies (4). Thus, the need to further investigate the pathogenesis of AD is critical. The major pathological features of AD include an enlarged and degenerative medial layer, vascular smooth muscle cell (VSMC) death and loss, and collagen-elastic fiber crosslinking disorder and fragmentation (5–7). Given that elastin (ELN) and collagen are two of the most abundant extracellular matrix (ECM) proteins, their adequate crosslinking is necessary to maintain the vessel's normal function (8). Moreover, mutations in ECM-associated genes [e.g., fibrillin 1 (FBN1) and lysyl oxidase (LOX)] lead to the occurrence of AD (9, 10).

Lysyl oxidase and its family members are the primary enzymes that regulate the crosslinking of ELN and collagen (11). The LOX family, including LOX, LOX-like 1 (LOXL1), LOXL2, LOXL3, and LOXL4, consists of mammalian copper-binding amine oxidases (12). These enzymes oxidize lysine and hydroxylysine ε-amino groups in ELN and collagen into highly reactive aldehydes, which then spontaneously react to form a variety of inter- and intra-chain crosslinks (8, 11). Lysyl oxidase family members (LOXs) play crucial roles in the cardiovascular system. For example, *Lox*^−/−^ mice died perinatally due to severe vascular abnormalities (e.g., aortic aneurysms and aortic tortuosity) (13); *Loxl1*^−/−^ mice were also found to have vascular defects (14). More importantly, accumulated evidence suggests that *LOX* mutations, such as p.Met292Arg, p.Met298Arg, p.Ser280Arg, and p.Ser348Arg, are closely associated with aortic dilation, thoracic aortic aneurysm and AD in humans (8, 10, 15). However, conflicting results on the role of LOX in vasculopathy in patients with Marfan syndrome (MFS) have been reported. Some studies reported that LOX expression and activity were reduced in the aortas of MFS patients, while other research demonstrated that comparable LOX mRNA levels and activity were found in the aortas of MFS patients and healthy subjects (11). Intriguingly, O Busnadiego et al. showed that the expression levels of LOX and LOXL1 were increased in the aortas of MFS patients and in a MFS mouse model (*Fbn1*^C1039G/+^) (11). Thus, the need to clarify the expression pattern of the LOX family, as well as their function in aortic dilation, is urgent. Aortic dilation and MFS are risk factors or etiologies of AD, but the expression patterns and roles of the LOX family in AD, especially non-hereditary AD, remain largely unknown. In addition, recent findings have shown that the functions of LOXs extend beyond their role in stabilization of the ECM and include roles in cell chemotactic responses, cell proliferation, adhesion, migration, and transcriptional regulation (13). Thus, investigating the non-classical functions of the LOX family in VSMCs would be interesting.

Here, we show that the protein levels of LOXL2 and LOXL3 are elevated in the aortas of patients with TAAD, but LOXL2 and LOXL3 play different roles in VSMCs. LOXL2 affects ECM by upregulating MMP2, while LOXL3 facilitates VSMC proliferation. Furthermore, the inhibitory effects of metformin and losartan on aortic dilatation may be partially achieved by suppressing LOXL2 and LOXL4 expression. These results suggest that activating LOXL3 and inhibiting LOXL2 might be a novel therapeutic strategy for the prevention of AD.

## Methods

### Human Aorta Samples

The human thoracic aortic tissue samples were collected from Division of Cardiothoracic and Vascular Surgery at Tongji Hospital, Tongji Medical College, Huazhong University of Science and Technology, with approval of Institutional Review Board. Written informed consent was obtained from all subjects. All experiments conducted with human tissue samples were performed in accordance with the relevant guidelines and regulations. Samples were obtained from patients with TAAD and donors for cardiac transplant. We did not conduct genetic testing on all patients, so we were unable to rule out whether these patients had unknown mutations in genes. Detailed patient information is displayed in Supplementary Table 1.

### Gene Expression Profiles in the Gene Expression Omnibus Database

We used the keywords “aortic dissection” and “Homo sapiens [porgn]” to search datasets in the GEO database (https://www.ncbi.nlm.nih.gov/geo/). To investigate the expression levels of LOXs in patients with AD, we included all microarray and RNA-sequence datasets with both control and AD samples. Thus, four gene expression datasets, “GSE153434,” “GSE98770,” “GSE52093,” and “GSE147026,” were included in the present study. The GSE153434 dataset contains data from 10 TAAD samples and 10 control samples and is based on the GPL20795 platform [HiSeq X Ten (*Homo sapiens*)]. The GSE98770 dataset contains data from six TAAD samples and five control samples and is based on the GPL14550 platform [Agilent-028004 SurePrint G3 Human GE 8×60K Microarray (Probe Name Version)]. The GSE52093 dataset contains data from seven TAAD samples and five normal samples and is based on GPL10558 (Illumina HumanHT-12 V4.0 Expression BeadChip). The GSE147026 dataset, which contains four aorta tissue samples from donors and four aorta tissue samples from patients with AD, is based on GPL24676 [Illumina NovaSeq 6000 (*Homo sapiens*)].

### Western Blot and Antibodies

Total proteins in human aortic smooth muscle cell (HASMC) and aortic tissues were extracted by RIPA solution with phosphatase inhibitors and protease inhibitors, and western blot was performed as described previously (3, 16, 17). The antibodies used in this study included α-SMA (ab7817), LOX (ab174316), SM22 (ab14106), H3K4me1 (ab8895), H3K36me3 (ab9050), Bax (ab32503), and Bcl-2 (ab182858) which were bought from Abcam. LOXL1 (A10191) and LOXL4 (A13131) were got from ABclonal. LOXL2 (GTX105085), MMP2 (GTX634832), MMP9 (GTX100458), H3K9me3 (GTX121677), and PCNA (GTX100539) were purchased from GeneTex. LOXL3 (sc-377216) was obtained from Santa Cruz. β-actin (#8457S), Beclin-1 (#3495), S6 (#2317), phosphorylation of S6 (p-S6) (#5364), AKT (#4685), phosphorylation of AKT (p-AKT) (#4060), p38 (#8690), p-p38 (#4511), p-JNK1/2 (#9255), p-ERK1/2 (#4370), p-GSK3 β (#5558), H3K4me2 (#9725), H3K4me3 (#9727), p-p65 (#3033), cyclinD1 (#2978), and LC3 (#12741) were purchased from Cell Signaling Technology.

### Real-Time PCR

Total mRNA was extracted from human aorta tissues and HASMCs by using TRI Reagent® solution (AM9738; ThermoFisher Scientific). The precipitated mRNA was dissolved in nuclease-free water, and the RNA concentration was determined by a Nanodrop2000 (ThermoFisher Scientific). Subsequently, mRNA was reverse transcribed into cDNA by using a Transcriptor First Strand cDNA Synthesis Kit (4896866001, Roche). Then, 2 μl of the cDNA solution, primers, DEPC water and Hieff® qPCR SYBR® Green Master Mix (YEASEN, 11201ES08) were added to each well for the real-time PCR assay. Furthermore, the relative mRNA levels were detected by the CFX Connect™ Real-Time PCR Detection System (Bio-Rad). The primers used in this study are listed in Supplementary Table 2.

### Plasmids

LOXL2 and LOXL3 knockdown plasmids were constructed by cloning double-stranded shRNA oligonucleotides targeting human LOXL2 and LOXL3 into the pLKO.1 plasmid at AgeI and EcoRI restriction enzyme sites. The targeting sequences of shRNA were shLOXL2: CCAGATAGAGAACCTGAATAT; shLOXL3: CATCTTCACTCACTATGATAT.

### Primary Human Aorta Smooth Muscle Cell Culture and Treatments

Ascending aortic tissues were taken from patients who underwent heart transplantation. Then, the tissues were placed into culture dishes filled with Dulbecco's modified Eagle's medium (DMEM)/F12 (SH30023.01; HyClone) at 4°C. Intimal and residual adventitial tissues were stripped under a stereomicroscope. Subsequently, the middle layer of the aortic wall was cut into small pieces (1–2 mm) and transferred to cell culture flasks with 5 ml of DMEM/F12 supplemented with 10% fetal bovine serum (SH30084.03; HyClone) and 1% penicillin-streptomycin (15140-122; ThermoFisher Scientific). A few days later, long, spindle-shaped HASMCs were observed and then passaged when the degree of cell fusion reached approximately 80%. Human aortic smooth muscle cells were cultured at 37°C in a humidified incubator with 5% CO_2_. Human aortic smooth muscle cells were infected with lentivirus containing shLOXL2 or shLOXL3 to knockdown LOXL2 or LOXL3 according to our previously reported methods (3, 17). Human aortic smooth muscle cells were starved for 12 h and then treated with losartan (10 and 100 μM, S1359; Selleck) and metformin (5 and 10 mM, HY17471A; MedChemExpress) for 48 h, with DMSO treatment serving as a control.

### EdU Incorporation Assay

Human aortic smooth muscle cells infected with lenti-PLKO.1 or lenti-shLOXL3 were plated in 24-well plates and incubated with EdU medium (50 μmol/L) for 4 h. After washing with phosphate-buffered saline (PBS) twice, HASMCs were fixed with 4% paraformaldehyde for 30 min. Then, the HASMCs were incubated with glycine (2 mg/ml) to neutralize paraformaldehyde and immersed in 0.5% Triton X-100 for 10 min. The HASMCs were stained with 1× Apollo staining solution, and the nuclei were stained with 1× Hoechst 33342 for 30 min at room temperature. Images were captured with a BX53 Olympus light microscope system.

### Statistical Analyses

All the data are represented as the mean ± standard deviation (*SD*) in the present study. For bioinformatics analysis, because different platforms were used for each dataset, we analyzed each dataset separately. We downloaded data from the supplementary file and selected raw data instead of the processed matrix file. To analyze the data, the raw expression values in each dataset were normalized by the R package limma, and log_2_ conversion was completed. After processing, we calculated the *p*-value for the difference in expression between AD samples and control samples by Student's *t*-test. Statistical analyses to compare the means of two groups were carried out by using Student's two-tailed *t*-test, while multiple group comparisons were conducted by using one-way ANOVA with least significant difference (equal variances assumed) or the Tamhane T2 (equal variances not assumed) test in SPSS software (version 13.0). *p* < 0.05 was used to indicate a statistically significant difference.

## Results

### Analysis of LOXs Expression Levels in the Aorta Based on Data From the Gene Expression Omnibus Database

**Figure 1:**
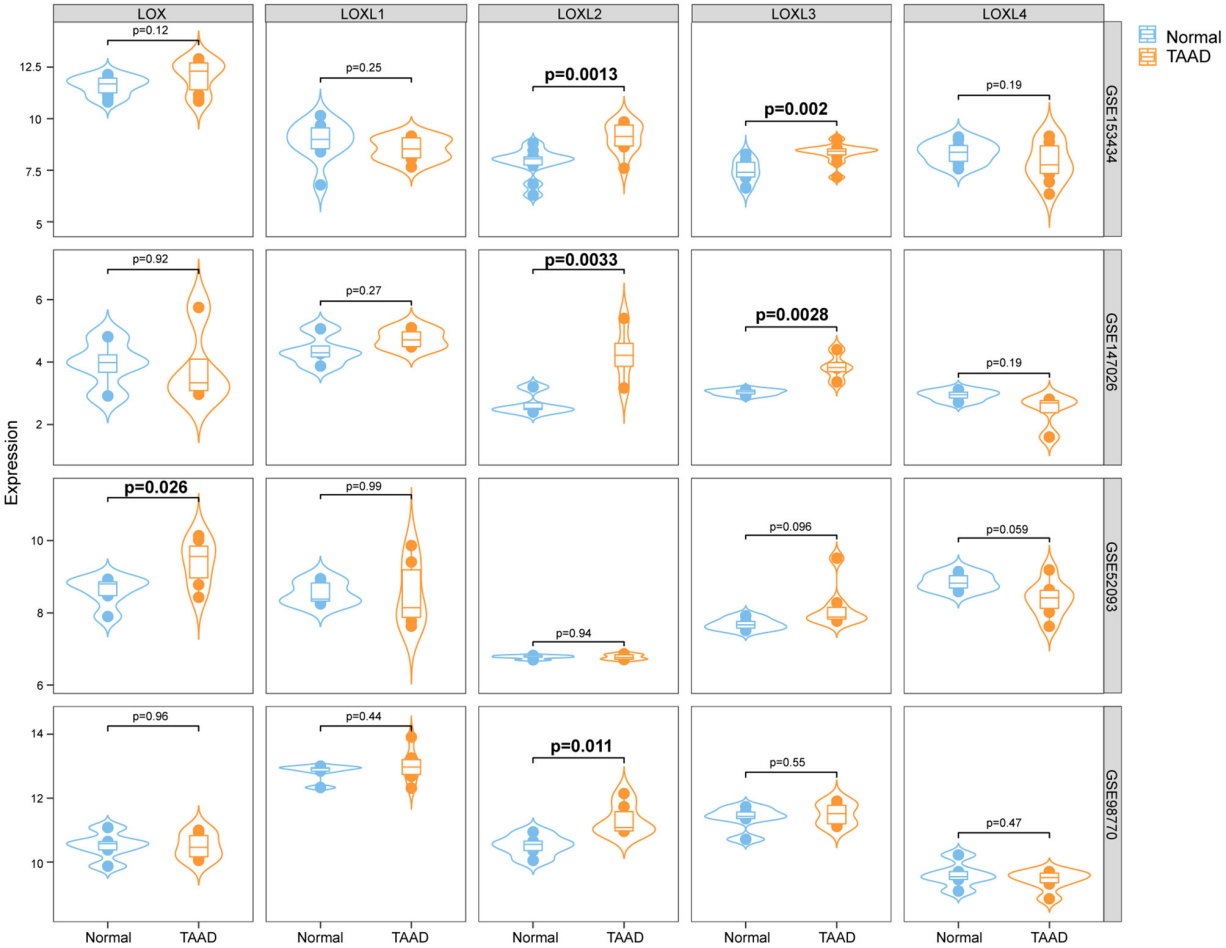
The relative mRNA levels of LOXs in the aortas of individuals with or without AD. We downloaded four datasets from the GEO database, and the relative mRNA levels of LOXs in the GSE153434, GSE147026, GSE52093, and GSE98770 datasets are shown. Normal indicates aorta samples from individuals without aortic dissection, and TAAD indicates aorta samples from individuals with Stanford type A aortic dissection.

To clarify the role of LOXs in AD, we first analyzed the relative mRNA expression levels of LOXs in aorta samples from patients with TAAD and normal aorta samples from GEO datasets (GSE153434, GSE147026, GSE52093, and GSE98770). These four datasets include a total of 24 normal and 27 TAAD samples. Because different platforms were used to prepare the datasets, the expression levels of LOXs in four datasets are shown individually in Figure 1. Specifically, in the GSE153434 and GSE147026 datasets, the mRNA levels of LOXL2 and LOXL3 were significantly increased in the aortas of TAAD patients. In the GSE52093 dataset, elevated LOX mRNA levels were detected in individuals with TAAD, and in the GSE98770 dataset, LOXL2 expression levels were higher in samples from patients with TAAD than in those from their normal counterparts. However, no significant changes in the expression of other LOX family members were detected (Figure 1). These results indicated that the occurrence of TAAD is accompanied by expression changes in specific LOXs, but this finding needs further verification.

### Verification of the Expression Levels of LOXs in the Human Aorta

**Figure 2:**
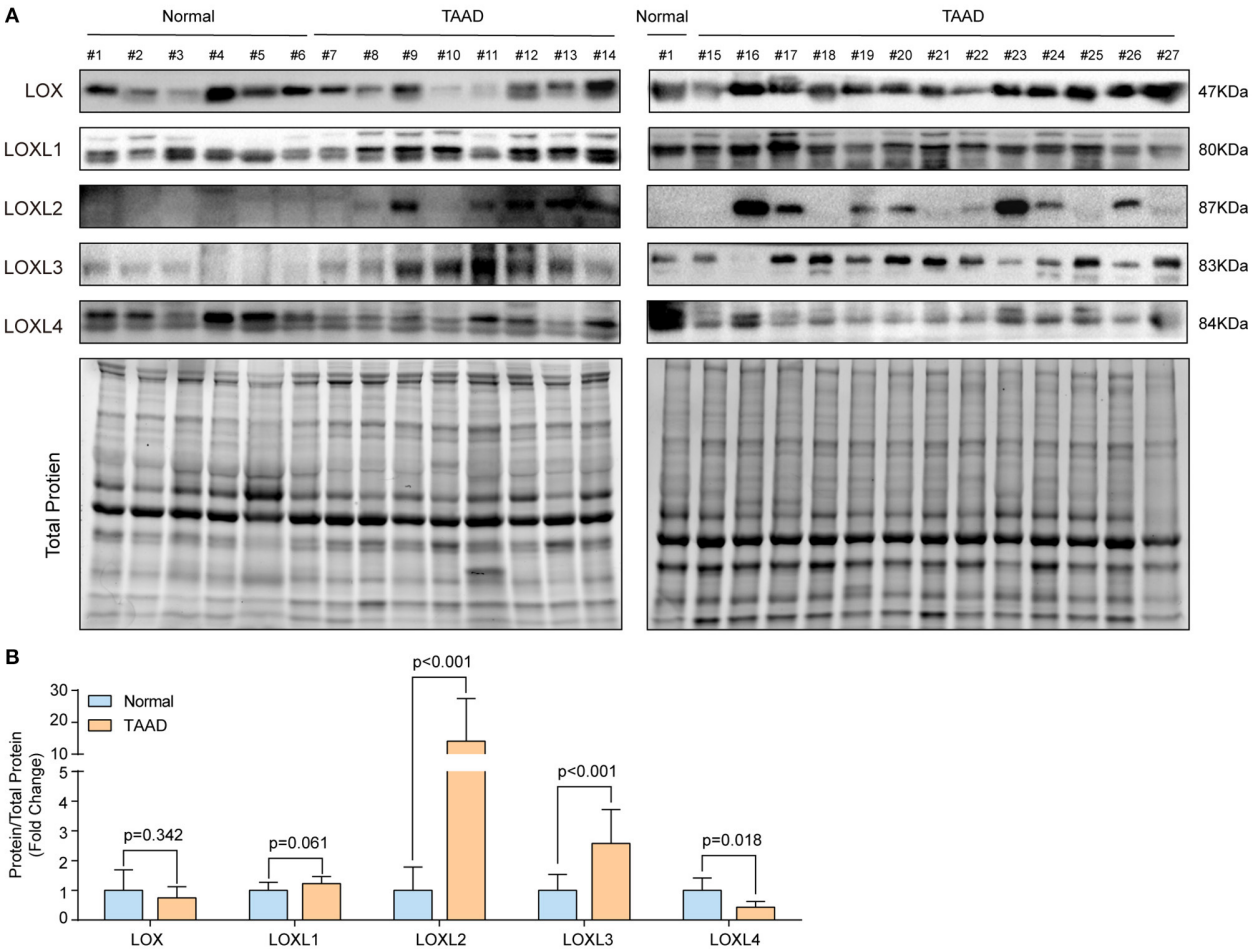
The protein levels of LOXs in the human aorta. **(A)** The protein levels of LOXs were detected in aorta samples from 6 normal controls and 21 TAAD patients by using western blotting. **(B)** Relative quantification of LOXs protein levels in **(A)**. Total protein served as the loading control.

Because the four GEO datasets were collected from a relatively small sample size and no consistent conclusions were drawn, we further verified the protein levels of LOXs in aorta samples from normal controls and TAAD patients in our own center. The detailed demographic and clinical characteristics of the patients are presented in Supplementary Table 1. Compared with those in normal controls, the protein levels of LOXL2, and LOXL3 were significantly increased, while LOXL4 protein levels were decreased in patients with TAAD, but comparable protein levels of LOX and LOXL1 was observed between groups (Figures 2A,B). We further investigated the relationship between LOXs expression levels and aortic diameter in 33 TAAD patients. The results demonstrated that except for the significant correlation between LOXL1 mRNA expression and the diameter of the aortic arch, other LOXs showed no significant correlation with the diameter of the ascending aorta, aortic arch, descending aorta, or abdominal aorta (Supplementary Figures 1A–E).

### LOXL2 Regulates ECM in Primary Cultured HASMCs

**Figure 3:**
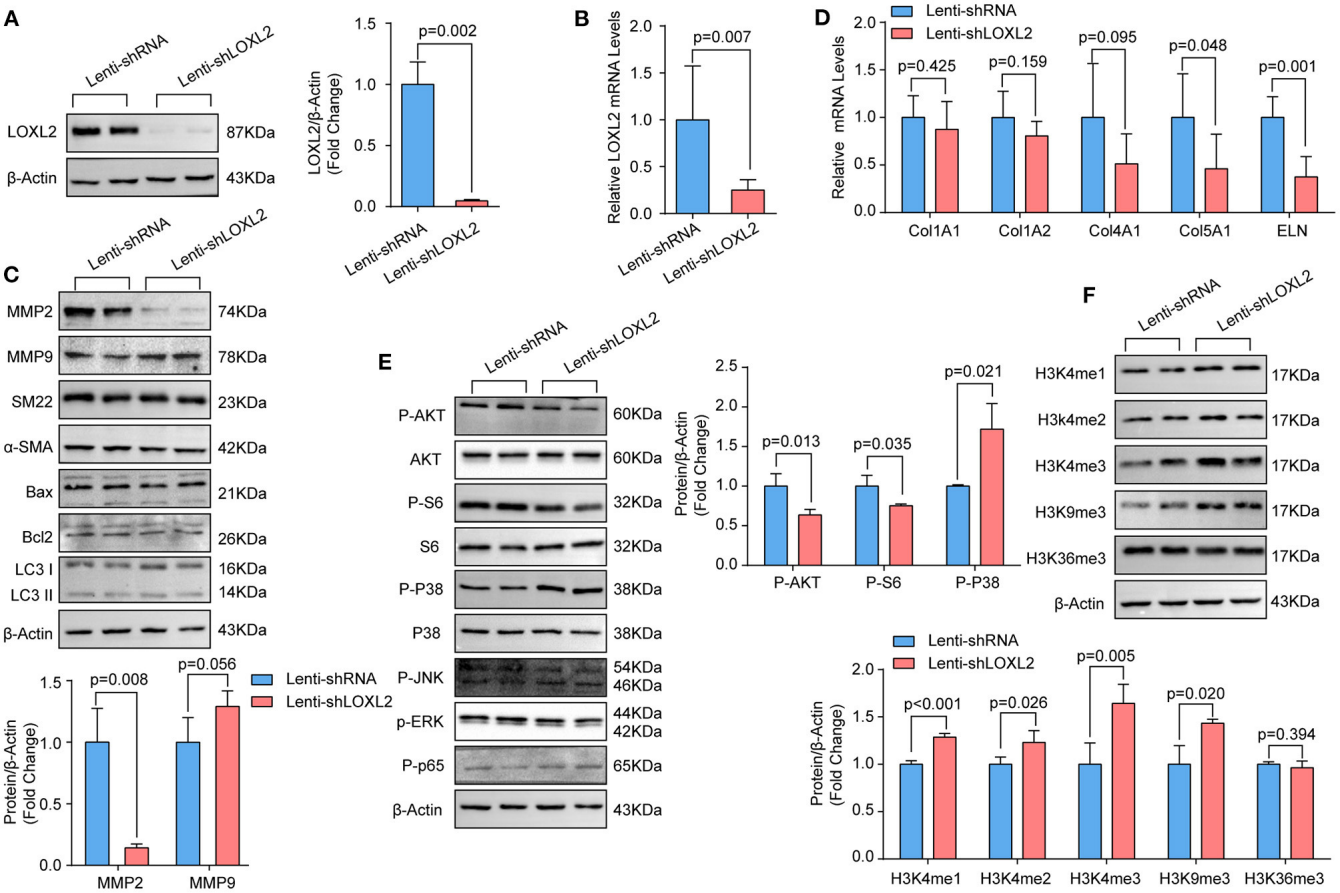
Knockdown of LOXL2 inhibited MMP2 expression and AKT signaling, but facilitated the methylation of H3K4 and H3K9 in primary cultured HASMCs. **(A)** The protein levels of LOXL2 were evaluated by western blotting (*n* = 4). **(B)** The mRNA levels of LOXL2 in HASMCs infected with lenti-shRNA, lenti-shLOXL2 were detected by RT-PCR (*n* = 5). **(C)** The protein levels of MMP2, MMP9, SM22, Bax, Bcl2, and LC3I/II in HASMCs infected with lenti-shRNA or lenti-shLOXL2 (*n* = 4). **(D)** The mRNA levels of Col1A1, Col1A2, Col4A1, Col5A1, and ELN in HASMCs were detected by RT-PCR (*n* = 5). **(E)** The p-AKT, S6, GSK3β, p38, ERK1/2, JNK1/2, and p65 in HASMCs was evaluated by western blotting (*n* = 4). **(F)** Western blotting was used to detect the protein levels of H3K4me1, H3K4me2, H3K4me3, H3K9me3, and H3K36me3 in HASMCs infected with lenti-shRNA or lenti-shLOXL2 (*n* = 4). β-Actin served as the loading control.

In human TAAD samples, we found that LOXL2 protein levels were obviously upregulated. Given that LOXL2 is critical for ECM regulation, we first evaluated the impact of LOXL2 knockdown on ECM. We constructed short hairpin RNA (shRNA) targeting LOXL2, which was packaged into lentivirus and then used to infect primary cultured HASMCs to knock down LOXL2. The LOXL2 mRNA and protein levels were verified, the results of which demonstrated that the mRNA and protein levels of LOXL2 were remarkably reduced in HASMCs infected with shLOXL2 (Figures 3A,B). More importantly, we found that the protein level of MMP2, but not MMP9, was dramatically reduced after LOXL2 knockdown in HASMCs (Figure 3C). Given that MMP2 is the primary matrix metalloproteinase that regulates Col1A1, Col1A2, Col4A1, Col5A1, and ELN (18), our results showed that Col5A1 and ELN were downregulated by LOXL2 knockdown, while Col1A1, Col1A2, and Col4A1 were not affected by LOXL2 deficiency (Figure 3D). These results indicates that LOXL2 plays a critical role in ECM regulation by promoting MMP2, Col5A1, and ELN expression.

The phenotypic switching of HASMCs is one of the features of AD (19). However, LOXL2 knockdown did not affect expression of the contractile markers α-SMA or SM22 (Figure 3C). Apoptosis and autophagy were reported to affect HASMC loss during AD (3, 17, 20). However, our data showed that LOXL2 knockdown had no impact on the expression of the apoptosis markers Bcl2 or Bax, or the autophagy marker LC3I/II (Figure 3C).

### LOXL2 Knockdown Inhibited AKT Phosphorylation but Activated p38 in Primary Cultured HASMCs

LOXL2 knockdown was reported to inhibit p38 phosphorylation in MDA-MB-231 cells (21), and LOXL2 accelerates cardiac fibroblast-to-myofibroblast transformation by activating the PI3K/AKT signaling pathway to produce TGFβ2 (22). Thus, we wondered whether the p38 and AKT signaling pathways would be regulated by LOXL2 in HASMCs. Our results demonstrated that knockdown of LOXL2 suppressed AKT and S6 ribosomal protein phosphorylation but facilitated p38 phosphorylation in HASMCs (Figure 3E). However, comparable levels of phosphorylated JNK1/2 and ERK1/2 were detected in HASMCs with or without LOXL2 knockdown (Figure 3E). In addition, LOXL2 inhibits NF-κBp65 phosphorylation in ATDC5 cells, and NF-κBp65 activation is also involved in AD (23). Unexpectedly, the phosphorylation of NF-κBp65 was not regulated by LOXL2 in HASMCs (Figure 3E).

### LOXL2 Inhibited H3K4me1/2/3 and H3K9me3 in HASMCs

Our previous data demonstrated that histone methylation is involved in the pathologic process of TAAD in humans (6). Recently, Peiró et al. found that LOXL2 could oxidize histone H3 at lysine 4 (H3K4ox) to prevent the di- and trimethylation in H3K4 but had no effect on H3K9me2 or H3K9me3 (24, 25). Thus, we further detected the levels of H3K4me1/2/3, H3K9me3, and H3K36me3 in HASMCs with LOXL2-knockdown. We found that H3K4me1/2/3 and H3K9me3 were significantly upregulated after LOXL2 knockdown, while H3K36me3 were not affected by LOXL2 (Figure 3F).

### Knockdown of LOXL3 Inhibited Primary Cultured HASMCs Proliferation

**Figure 4:**
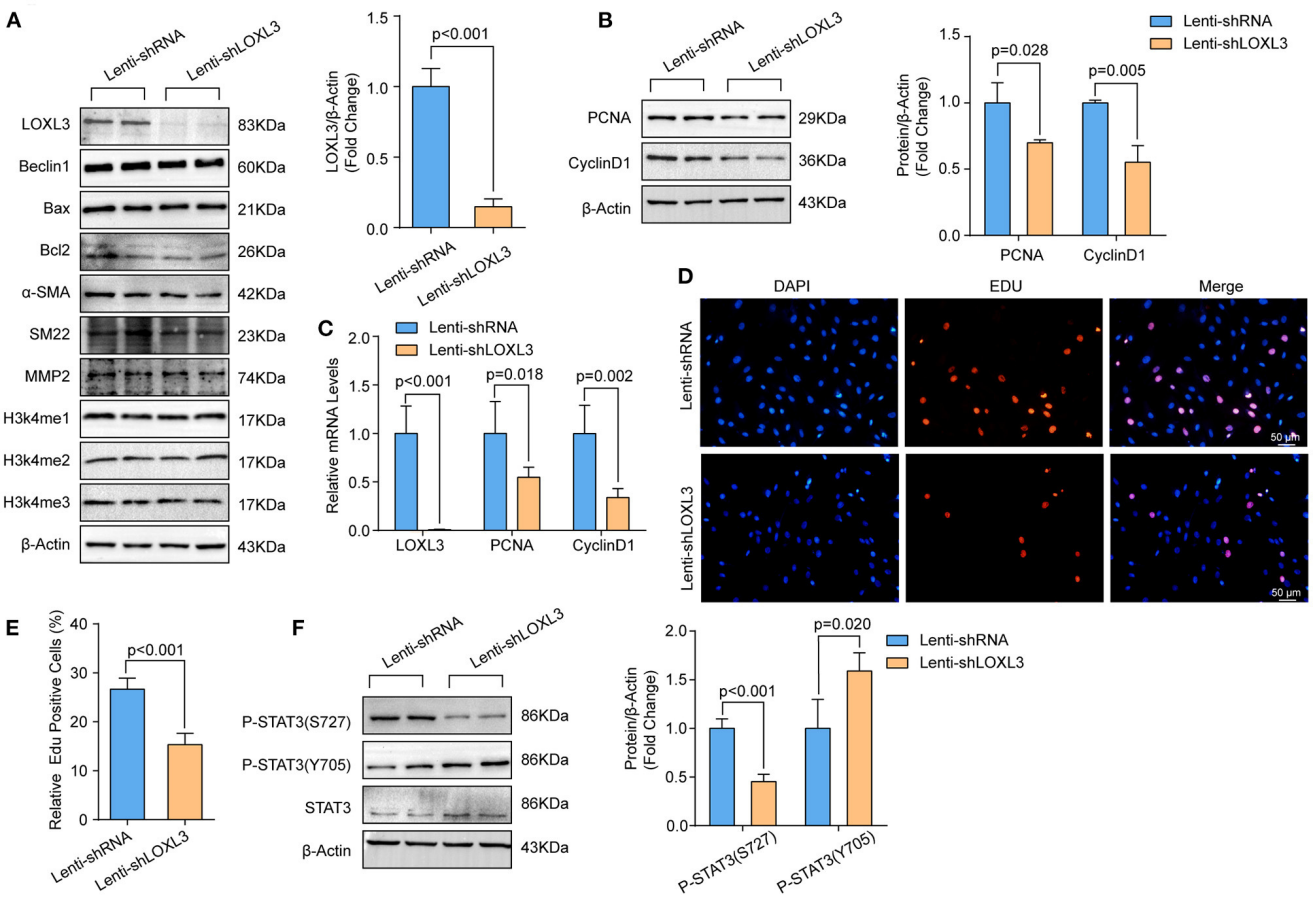
LOXL3 knockdown suppressed the proliferation of primary cultured HASMCs. **(A)** The protein levels of LOXL3, Beclin 1, Bax, Bcl2, α-SMA, SM22, MMP2, H3K4me2, and H3K4me3 in HASMCs infected with lenti-shRNA or lenti-shLOXL3 (*n* = 4). **(B)** The protein levels of the proliferation markers cyclin D1 and PCNA in HASMCs infected with lenti-shRNA or lenti-shLOXL3 (*n* = 4). **(C)** The mRNA levels of LOXL3, cyclin D1, and PCNA in the indicated HASMCs were evaluated by RT-PCR (*n* = 5). **(D,E)** The EdU assay was performed in HASMCs infected with lenti-shRNA or lenti-shLOXL3 **(D)**, and EdU-positive cells were quantified **(E)** (*n* = 4). **(F)** Levels of STAT3 phosphorylated at serine 727 and tyrosine 705 in HASMCs infected with lenti-shRNA or lenti-shLOXL3 (*n* = 4). β-Actin served as the loading control.

To investigate the role of LOXL3 in HASMCs, we first knocked down LOXL3 in primary cultured HASMCs (Figures 4A,C). We subsequently evaluated the impact of LOXL3 on autophagy, apoptosis, and phenotypic switching, and the results indicated that LOXL3 knockdown had no effect on these biological processes, as evidenced by the similar protein levels of Beclin-1, Bax, Bcl2, α-SMA, and SM22 in HASMCs with or without LOXL3 knockdown (Figure 4A). Additionally, LOXL3 did not regulate MMP2 expression or H3K4 methylation (Figure 4A). Intriguingly, we found that PCNA and cyclin D1 were downregulated by LOXL3 knockdown in HASMCs, indicating that proliferation had been inhibited (Figures 4B,C). Furthermore, the EdU incorporation assay indicated that EdU-positive HASMCs were remarkably reduced after LOXL3 knockdown (Figures 4D,E). STAT3 is a ground-state transcription factor required for cell proliferation, and LOXL3 deacetylates/deacetyliminates STAT3, disrupting its dimerization, nuclear translocation, and activation to restrict cell proliferation (26). However, we found that LOXL3 knockdown facilitated the phosphorylation of Tyr705 in STAT3, and inhibited the phosphorylation of Ser727 in STAT3 in HASMCs (Figure 4F). These results indicated that LOXL3 is a positive regulator that accelerates HASMC proliferation.

### The Effect of Losartan and Metformin on LOXs Expression

**Figure 5:**
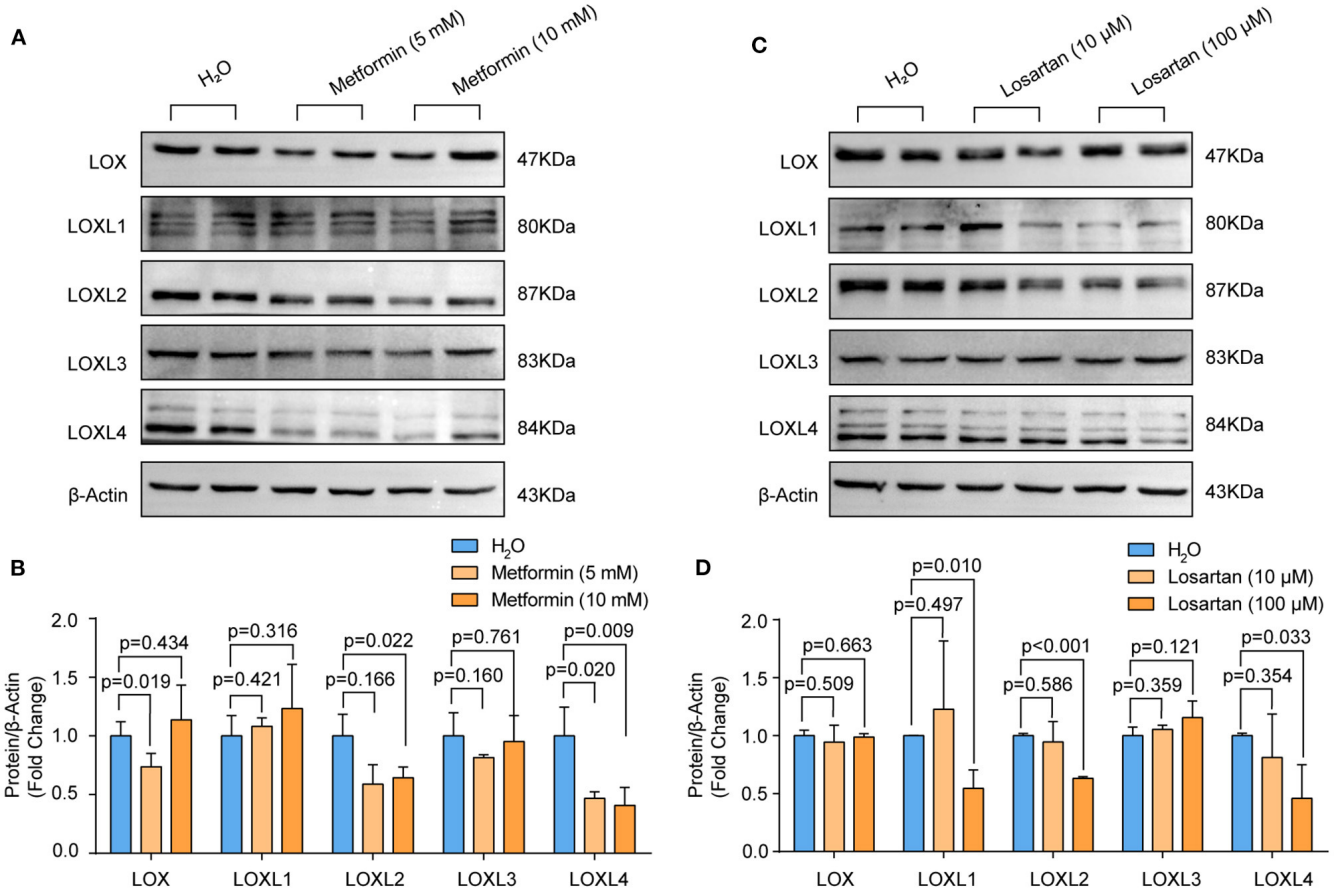
The protein levels of LOXs in primary cultured HASMCs treated with metformin or losartan. **(A,B)** The protein levels of LOX, LOXL1, LOXL2, LOXL3, and LOXL4 in HASMCs stimulated with 5 or 10mM metformin were evaluated by western blotting (*n* = 4). **(C,D)** LOX, LOXL1, LOXL2, LOXL3, and LOXL4 protein levels in HASMCs treated with 10 or 100 μM losartan were detected by using western blotting (*n* = 4). H_2_O-treated HASMCs served as the control group in **(A)** and **(B)**.

Accumulating evidence has demonstrated that losartan and metformin have the potential to delay expansion of the aortic root or abdominal aorta or even prevent this major life-threatening manifestation in patients with MFS or AAA (27, 28). Thus, we wondered whether LOXs are involved in the effect of losartan and metformin on the aorta. We treated primary cultured HASMCs with metformin (5 and 10 mM) or losartan (10 and 100 μM) for 48 h. Our results showed that the protein levels of LOX (5 mM), LOXL2 and LOXL4 were reduced in HASMCs treated with metformin, while LOXL1 and LOXL3 were not affected by metformin (Figures 5A,B). Instead, losartan inhibited LOXL1, LOXL2, and LOXL4 expression at a concentration of 100 mM, but not LOX or LOXL3 expression in HASMCs (Figures 5C,D). These results suggest that LOXL2 and LOXL4 may partially mediate the protective roles of both losartan and metformin on aorta dilation and AAA.

## Discussion

Lysyl oxidases are well known to be crucial for ECM (13). However, the expression levels of LOXs in the aortas of patients with TAAD and their non-canonical functions in VSMCs are unclear. In the present study, we found that the protein levels of LOXL2 and LOXL3 are upregulated in the aortas of TAAD patients. Knockdown of LOXL2 inhibited MMP2, p-AKT, and p-S6 but promoted H3K4me1/2/3, H3K9me3, and p-P38 in HASMCs, which may be involved in regulating ECM. Instead, LOXL3 knockdown suppressed cyclin D1 and PCNA expression to inhibit HASMC proliferation. Moreover, LOXL2 and LOXL4 may partially mediate the therapeutic effects of losartan and metformin on aortic dilatation. Therefore, our present study not only detected the expression patterns of LOXs in patients with TAAD but also clarified the functions of LOXL2 and LOXL3 in HASMCs.

Lysyl oxidases are the enzymes responsible for the crosslinking between collagen and ELN, which is critical to the structural integrity of the aorta (13). Accumulating evidence has demonstrated that the mutation or downregulation of LOX is closely related to aorta dilation and AD (15), and knockout of LOX in mice resulted in perinatal death due to AD rupture and was accompanied by reductions in ELN- and collagen-specific crosslinks in the aorta by 60 and 40%, respectively (29). BAPN is an irreversible inhibitor of LOX activity that can efficiently induce AD in both young mice and rats, but it is less efficient in adult mice and rats (7). The reason for this phenomenon may partially be because that ELN layers in the aorta are laid down during development and have a long half-life (approximately 40 years) (10, 30). In addition, a high-fat diet induced LOX overexpression in the aortas of rats, and BAPN prevented an increase in circulating leptin levels, reducing fibrosis, in rats fed a high-fat diet (31). Lysyl oxidases was also found to be upregulated in the vitreous of diabetic subjects (32). Given that diabetes mellitus is negatively associated with AD (33), diabetes-induced LOX overexpression in the aorta may mediate this protective effect.

In addition to LOX, the roles of other members of the LOX family in AD have been rarely reported. In the present study, we found that LOXL2 and LOXL3 protein levels are enhanced, while the protein level of LOXL4 is reduced in patients with TAAD compared to normal subjects. Although LOXL2 has been widely studied in cancer and fibrotic diseases such as idiopathic pulmonary fibrosis, liver fibrosis, and cardiac fibrosis (34), its expression and function in AD were unclear. Our results showed that compared with that in non-AD aorta samples, the protein level of LOXL2 in the aortas of patients with TAAD was remarkably increased. Wong et al. reported that chronic inflammation could induce LOXL2 expression and extracellular secretion in the liver (35, 36). The pathological process of AD is accompanied by inflammation, and excessive inflammation is an independent predictor of adverse clinical outcomes (37). Thus, elevated LOXL2 expression levels in the aortas of AD patients may be related to inflammation.

As LOXL2 is critical for ECM regulation (35), we detected the expression levels of MMP2 and MMP9 in primary cultured HASMCs with or without LOXL2 knockdown. Our results demonstrated that LOXL2 knockdown significantly suppressed MMP2 expression but not MMP9 expression. Increased MMP2 expression was also observed in the aorta of patients with AD (38). MMP2 is predominantly derived from VSMCs and fibroblasts, and MMP2 degrades collagen and ELN (38). Moreover, MMP2 deficiency significantly reduced BAPN-induced formation and AD rupture in mice (39). MMP2 deletion inhibited the activation of transforming growth factor-β (TGFβ) and its non-canonical signaling cascade downstream of extracellular signal regulated kinases 1/2 (ERK1/2) in the aortas of mice (40). Atorvastatin and amlodipine were reported to reduce MMP2 activity, and doxycycline inhibited MMP2 expression to delay aorta dilation (38, 40). Thus, LOXL2 may facilitate AD by inducing MMP2 expression. However, the precise effect of LOXL2 on AD and how it regulates the expression of MMP2 require further investigation.

We found that the expression levels of LOXL3 were elevated in the aortas of humans with AD. LOXL3 was initially identified as an amine oxidase, but a variety of novel functions have recently been attributed to LOXL3 (41). In our study, we demonstrated that LOXL3 deficiency in primary cultured HASMCs remarkably suppressed HASMC proliferation. As VSMC loss is one of the major feature of AD, the LOXL3-mediated regulation of VSMC proliferation may be a compensatory effect. In pancreatic ductal adenocarcinoma cells, LOXL3 physically interacted with SNAIL to promote proliferation (41). Another study demonstrated that LOXL3 interacted with proteins involved in DNA stability (e.g., BRCA2 and SMC1A) and mitosis completion to accelerate the proliferation of melanoma cells (42). However, LOXL3 colocalized with STAT3 in the nucleus to deacetylate STAT3, disrupt STAT3 dimerization, and then inhibit the transcriptional activity of STAT3 to restrict Th17 and Treg proliferation and differentiation (26). In our study, the levels of p-STAT3 (Ser727) were decreased, while those of p-STAT3 (Tyr705) were increased in LOXL3-knockdown HASMCs. It has been reported that the phosphorylation of Ser727 in STAT3 inhibits STAT3 dimerization and nuclear translocation by enhancing the dephosphorylation of Tyr705 in STAT3 (43). Thus, the impact of LOXL3 on VSMCs proliferation may not be associated with STAT3 phosphorylation, but whether LOXL3 regulates VSMCs proliferation by interacting with SNAIL, BRCA2 or SMC1A requires further research.

Accumulating evidence indicates that losartan (an AT1 antagonist) and metformin (a hypoglycemic drug) have an inhibitory effect on aortic dilation in MFS patients or AAA patients (27, 28, 44). A recently published long-term clinical study suggested that combined losartan and β-blocker treatment in patients with MFS had a clinical benefit (27). We treated primary cultured human VSMCs with losartan and metformin. Our results showed that both losartan and metformin inhibited LOXL2 and LOXL4 expression. Thus, LOXL2 and LOXL4 may be common targets of losartan and metformin in this condition. Nevertheless, other mechanisms are involved in their protective roles; for example, the PI3K/AKT/mTOR/autophagy pathway was inhibited by metformin to repress the pathophysiology of AAA (45).

Although we have systematically verified the expression of LOXs at the mRNA and protein levels in human clinical samples, we have not clarified the regulatory mechanisms that mediate these changes in LOXs expression. In addition, we studied the role of LOXL2 and LOXL3 at only the cellular level and did not use knockout animal models to reveal their roles in AD *in vivo*. In addition, whether LOXL1 and LOXL4 are also involved in the regulation of AD needs to be further explored.

## Conclusions

In conclusion, the present study demonstrated that increased LOXL2 in patients with TAAD might facilitate MMP2, Col5A1, and ELN expression to affect ECM in aorta. However, as LOXL3 knockdown inhibited VSMC proliferation, elevated LOXL3 seems to be a compensatory effect. Thus, the differential regulation of LOXs may be a novel strategy for the treatment of AD.

## Data Availability Statement

The datasets presented in this study can be found in online repositories. The names of the repository/repositories and accession number(s) can be found in the article/Supplementary Material.

## Ethics Statement

The studies involving human participants were reviewed and approved by the Human Research Ethics Committees of Tongji Hospital, Tongji Medical College, Huazhong University of Science and Technology. The patients/participants provided their written informed consent to participate in this study. The animal study was reviewed and approved by the Animal Care and Use Committee of Tongji Hospital, Tongji Medical College, Huazhong University of Science and Technology.

## Author Contributions

YZ, YC, and CL carried out the immunoassays and cell culture. XF, Z-MF, and JG performed the surgery and collected the samples. Z-MF, XY, and XJ participated in the design of the study and performed the statistical analysis. XY drafted the manuscript. XL revised the manuscript. XJ and Z-MF conceived of the study, participated in its design, and coordination and helped to draft the manuscript. XY and Z-MF provided funding support. All authors read and approved the final manuscript.

## Conflict of Interest

The authors declare that the research was conducted in the absence of any commercial or financial relationships that could be construed as a potential conflict of interest.

## Abbreviations

LOXs: lysyl oxidases
AD: aortic dissection
TAAD: Stanford type A AD
HASMCs: human aortic smooth muscle cells
VSMC: vascular smooth muscle cell
ECM: extracellular matrix
FBN1: fibrillin 1
LOXL1: LOX-like 1
MFS: Marfan syndrome
GEO: gene expression omnibus
PBS: phosphate-buffered saline
DMEM: Dulbecco's modified Eagle's medium
SD: standard deviation
shRNA: short hairpin RNA
ELN: elastin
TGFβ: transforming growth factor-β
ERK1/2: extracellular signal regulated kinases 1/2.
