# Supplementary material for: The Expression Patterns and Roles of Lysyl Oxidases in Aortic Dissection
Source: Front Cardiovasc Med. 2021 Jul 7;8:692856. doi: 10.3389/fcvm.2021.692856 (PMC8292648; doi:10.3389/fcvm.2021.692856)
Supplement: Supplementary file 1 [file Data_Sheet_1.pdf]

Supplemental Materials

Table S1. The clinical information of patients with TAAD

Clinical items	TAAD group (n=33)
Age (year)	49.91±7.67
Male [n (%)]	30 (90.91)
Smoking [n (%)]	15 (45.45)
Alcohol [n (%)]	7 (21.21)
Hypertension [n (%)]	18 (54.55)
Diabetes mellitus [n (%)]	3 (9.09)
Heart rate (bpm)	82.27±17.60
Systolic pressure (mmHg)	131.24±26.52
diastolic pressure (mmHg)	70.33±13.72
Diameter of aorta (mm)	
aortic arch (mm)	41.18±5.32
ascending aorta (mm)	48.81±6.94
thoracic aorta (mm)	35.36±4.38
abdominal aorta (mm)	30.10±2.62

The data are represented as the mean ± standard deviation (SD) or as percentage. TAAD: Stanford type A aortic dissection; Bpm: beats per minute;

Table S2. Real-time PCR primers

Primer names	Primers
Human-LOX forward primer	5'-GCATACAGGGCAGATGTCAGA-3'
Human-LOX reverse primer	5'-TTGGCATCAAGCAGGTCATAG-3'
Human-LOXL1 forward primer	5'-TGCCACCAGCATTACCACAG-3'
Human-LOXL1 reverse primer	5'-GAGGTTGCCGAAGTCACAGG-3'
Human-LOXL2 forward primer	5'-CTGCAAGTTCAATGCCGAGT-3'
Human-LOXL2 reverse primer	5'-TCTCCACCAGCACCTCCACTC-3'
Human-LOXL3 forward primer	5'-CGGATGTGAAGCCAGGAAACT-3'
Human-LOXL3 reverse primer	5'-AGGCATCACCAATGTGGCA-3'
Human-LOXL4 forward primer	5'-CAAAGGGTCCTGGGCAGAG-3'
Human-LOXL4 reverse primer	5'-ACACGACACTGGCAGAGATG-3'
Human-18S- forward primer	5'-CTCAACACGGGAAACCTCAC-3'
Human-18S- reverse primer	5'-CGCTCCACCAACTAAGAACG-3'
Human-PCNA- forward primer	5'-AGGTGTTGGAGGCACTCAAG-3'
Human-PCNA- reverse primer	5'-CGCGTTATCTTCGGCCCTTA-3'
Human-Cyclin D1- forward primer	5'-ATGCCAACCTCCTCAACGAC-3'
Human-Cyclin D1- reverse primer	5'-AAGACCTCCTCCTCGCACTT-3'
Human-COL1A2- forward primer	5'-TGAACTTGTTGCTGAGGGCA-3'
Human-COL1A2- reverse primer	5'-ACTGGGCCAATGTCCACAAA-3'

Figure S1

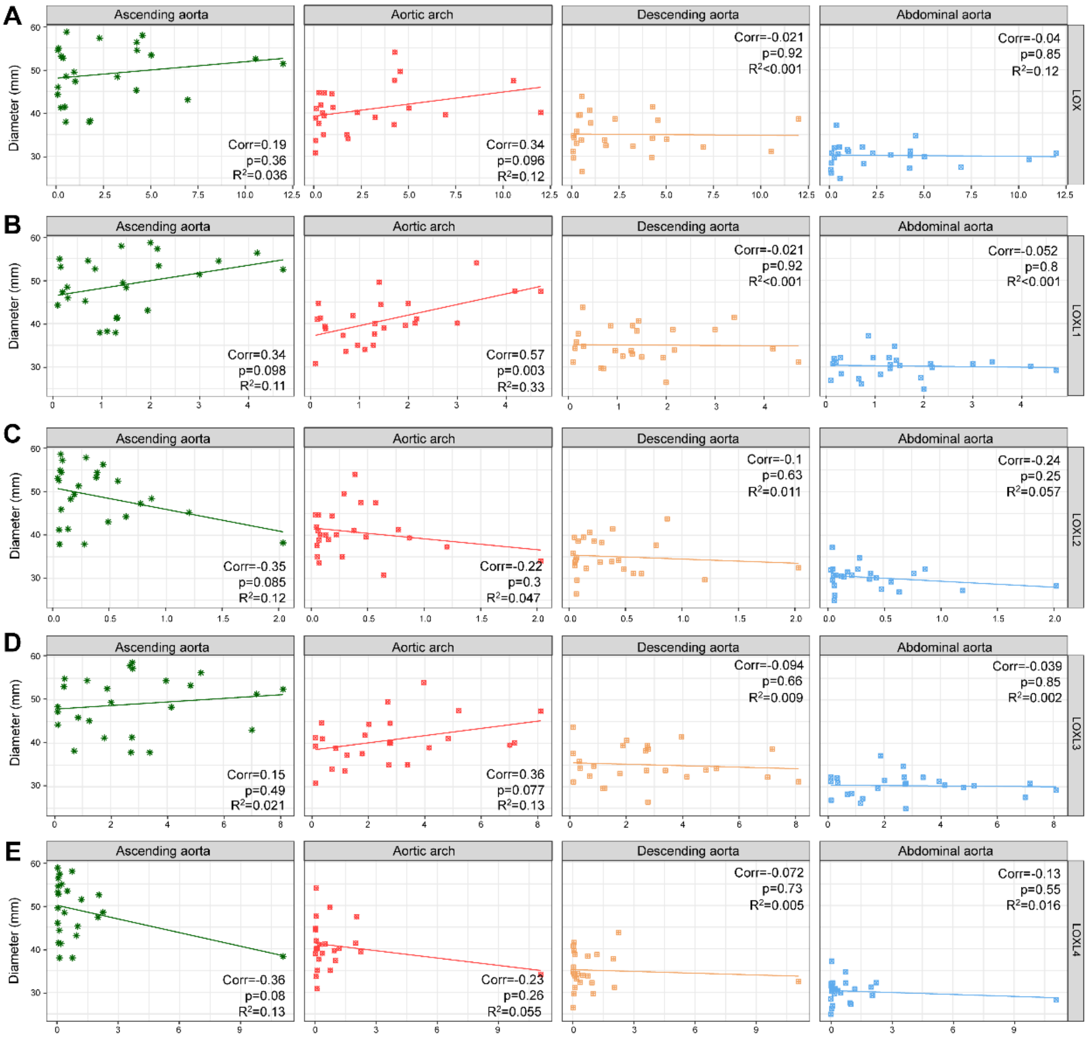

Figure S1. Correlation analysis between LOXs mRNA expression levels and aortic diameters. (A-E) Pearson correlation analysis was used to analyze the correlation between LOXs mRNA levels and the diameters of the ascending aorta, aortic arch, descending aorta and abdominal aorta.
